# High-Throughput Sequencing Reveals Tobacco and Tomato Ringspot Viruses in Pawpaw

**DOI:** 10.3390/plants11243565

**Published:** 2022-12-17

**Authors:** Jiyeong Choi, Anya Clara Osatuke, Griffin Erich, Kristian Stevens, Min Sook Hwang, Maher Al Rwahnih, Marc Fuchs

**Affiliations:** 1 School of Integrative Plant Science, Plant Pathology and Plant-Microbe Biology, Cornell University, Geneva, NY 14456, USA; 2 Cornell Cooperative Extension, Cornell University, Ithaca, NY 14853, USA; 3 School of Integrative Plant Science, Horticulture, Cornell University, Ithaca, NY 14853, USA; 4 Department of Plant Pathology, Foundation Plant Services, University of California, Davis, CA 95616, USA

**Keywords:** pawpaw, nepovirus, tobacco ringspot virus, tomato ringspot virus, high-throughput sequencing

## Abstract

Pawpaw (*Asimina triloba*) trees exhibiting stunting and foliar mosaic, chlorosis, or distortions were observed in New York. In 2021, leaf samples from two symptomatic trees and a sapling, as well as two asymptomatic trees, were tested for the presence of viruses and viroids by high-throughput sequencing (HTS) using total RNA after ribosomal RNA depletion. HTS sequence information revealed tobacco ringspot virus (TRSV) and tomato ringspot virus (ToRSV) in symptomatic but not in asymptomatic leaves. HTS reads and de novo-assembled contigs covering the genomes of both viruses were obtained, with a higher average read depth for RNA2 than RNA1. The occurrence of TRSV and ToRSV was confirmed in the original leaf samples used for HTS and 12 additional trees and saplings from New York and Maryland in 2022 by RT-PCR combined with Sanger sequencing, and DAS-ELISA. Single infections by TRSV in 11 of 14 trees and dual infections by TRSV and ToRSV in 3 of 14 trees were identified. The nucleotide sequence identity of partial gene fragments of TRSV and ToRSV was high among pawpaw isolates (94.9–100% and 91.8–100%, respectively) and between pawpaw isolates and isolates from other horticultural crops (93.6–100% and 71.3–99.3%, respectively). This study is the first to determine the virome of pawpaw.

## 1. Introduction

Pawpaw (*Asimina triloba*) is the only member of the neotropical family Annonaceae that is native to North America with wild populations found in forested lowland areas in Canada and the eastern United States, as well as the southern and midwestern United States. This fruit tree is considered a vulnerable or even an endangered species in several regions, including in New York state. Pawpaw trees have a pyramidal growth habit and may reach up to 10 m in height. They have a suckering habit with numerous saplings often surrounding adult trees [1]. Individual trunks live for approximately 40 years, but the root system can continue to generate new growth. Clonal saplings, also known as root suckers, originate from the root systems of mature trees. Pawpaw flowers are protogynous, and cross pollination from a genetically distinct tree, with a few cultivar exceptions is required to produce genetically heterogeneous seedlings arising from dropped seeds [1,2]. 

Pawpaw fruits are the largest edible fruit indigenous to North America. Blossoms emerge in May and have a pungent scent that is attractive to detritivore insects, including flies and beetles. The pawpaw flower has one to nine ovaries with each fertilized ovary gives rise to a large, many-seeded berry, fruit. Pawpaw fruits have a thin, pale green skin, and are borne singly or in clusters. The fruit is ripe between mid-September and October, depending on the growing site and the genotype. Ripe fruits possess a soft pulp that may be smooth, grainy, or gelatinous. Pawpaw trees reach their maximum fruit production after approximately seven years [3]. In several regions of the United States, there is an increased interest in pawpaw cultivation. For example, several groups of Indigenous Peoples in the territories surrounding the Great Lakes Region, including members of the Haudenosaunee Confederacy, are planting pawpaw as a traditional food crop [4,5].

An experimental orchard of pawpaw cultivars and advanced open-pollinated breeding lines was established at Cornell University in 1999. Virus-like symptoms consisting of tree stunting or foliar mosaic, patchy discolorations, chlorosis, mottling, and distortions were observed early on in some trees. In contrast, leaves of asymptomatic trees were uniformly dark green in color. Virus-like symptoms were apparent on trees and saplings, eventually suggesting the occurrence of a soil-borne agent. Symptoms were rarely present throughout an entire tree; instead, they were noticed along several adjacent branches with leaves on top of the scaffold of symptomatic trees often remaining asymptomatic, suggesting that some trees may have recovered from symptoms. Based on these observations, symptoms were assumed to be of virus origin.

In this study, we hypothesized that one or several viruses, perhaps soil-borne viruses for which plant recovery from symptoms is known, are present in symptomatic pawpaw trees. We used high-throughput sequencing (HTS) to characterize the virome of pawpaw trees. The HTS results were validated by RT-PCR in combination with Sanger sequencing and by serological assays.

## 2. Results

### 2.1. Virus-like Symptoms and Distribution of Symptomatic Trees in an Experimental Pawpaw Orchard 

**Figure 1:**
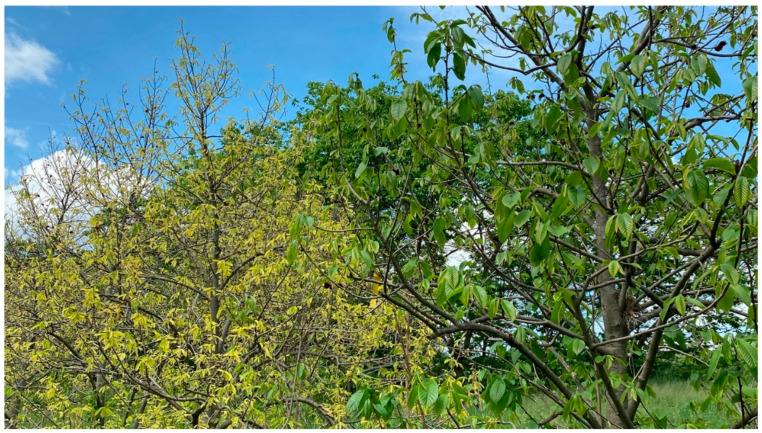
Symptomatic (left) and asymptomatic (right) pawpaw trees in an experimental orchard in Lansing, NY. The photo was taken in May 2022.

**Figure 2:**
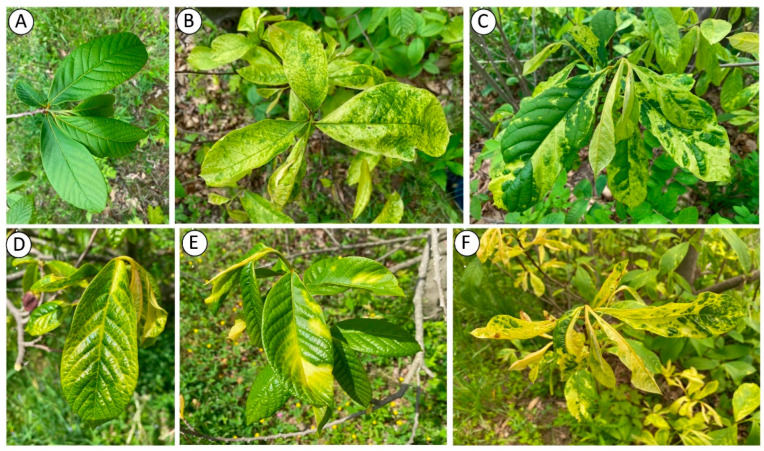
An asymptomatic leaf of a pawpaw tree (**A**) and foliar symptoms of discoloration (**B**), mosaic and distortion (**C**), vein clearing (**D**), patchy chlorosis (**E**), and chlorosis and distortion on pawpaw trees or saplings (**F**) in an experimental orchard in Lansing, NY. Photos were taken in May 2022.

**Figure 3:**
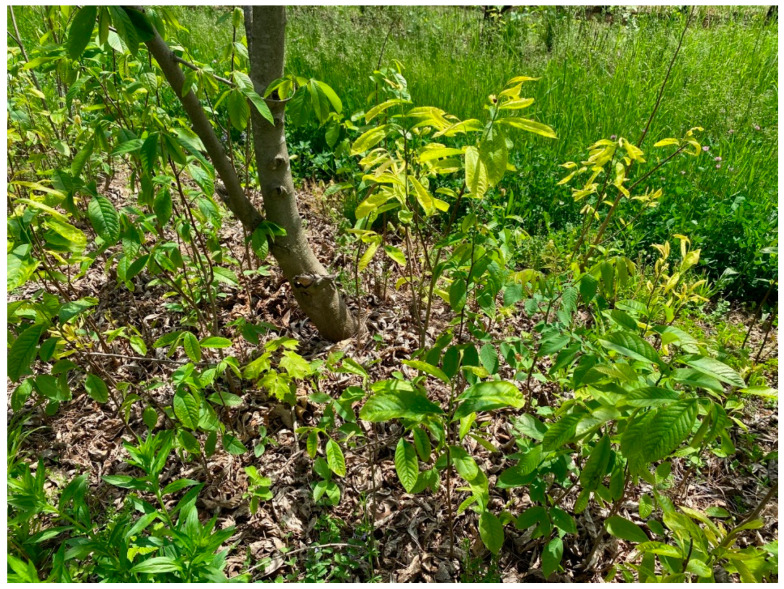
One- and two-year-old saplings growing underneath established pawpaw trees in an experimental orchard in Lansing, NY. Saplings may be root suckers or seedlings. Note that some saplings exhibit virus-like symptoms. The photo was taken in May 2022.

An experimental orchard of pawpaw cultivars and advanced open-pollinated breeding lines established at Cornell University in Lansing, New York in 1999 was selected for this study. Most trees and saplings exhibited virus-like symptoms in the experimental orchard. Prominent symptoms were tree stunting (Figure 1), leaf distortions, mosaic, vein clearing, patchy chlorosis, extended chlorosis, and mottling (Figure 2). Symptoms were often unevenly distributed in the tree canopy with asymptomatic leaves on some branches and in the upper tree scaffold. Monitoring pawpaw trees for virus-like symptoms in May 2022 revealed that 89% (199 of 224) of them were symptomatic. Trees from the 10 commercial pawpaw cultivars and 18 advanced open-pollinated seedling lines were symptomatic, suggesting no association between virus-like symptom presence and pawpaw genotype. Similar symptoms were apparent on trees and saplings surrounding the symptomatic trees with most saplings underneath symptomatic trees being symptomatic (Figure 3). 

A few asymptomatic trees (11%, 25 of 224) were identified. These were spatially located at the edge of the experimental orchard at the south end of rows 2 and 3 (6 and 10 trees per row, respectively), and north end of rows 1, 3, and 4 (3 trees per row). Saplings beneath the asymptomatic trees were predominantly asymptomatic.

### 2.2. Analysis of the Pawpaw Virome by HTS

To determine the occurrence of viruses in symptomatic pawpaw trees, four composite leaf samples were collected for nucleic acid isolation and HTS analysis following ribodepletion and cDNA library construction in 2021. Three composite leaf samples were from two symptomatic trees [tree 13 in row 1 (tree #1) and tree 5 in row 2 (tree #2)] and one symptomatic sapling growing underneath tree #1 in the experiment orchard. The fourth composite leaf sample was from two asymptomatic trees growing on the main campus of Cornell University in Ithaca, New York. 

HTS yielded between 16 and 24 million raw reads per cDNA library. Following trimming, contig assembly, and GenBank annotation, the total potential viroid and virus contigs totaled 354 for the four pawpaw samples. Analysis of assembled contigs identified sequences of one or two known viruses in the transcriptome of symptomatic samples but not in asymptomatic pawpaw trees: tobacco ringspot virus (TRSV) and tomato ringspot virus (ToRSV) (Table 1). TRSV and ToRSV are members of the species *Nepovirus nicotianae* and *Nepovirus lycopersici*, respectively, of the genus *Nepovirus* in the family *Secoviridae* [6]. No other contigs with at least 80% nucleotide or amino acid sequence similarity to other plant viruses for which sequences are available in NCBI GenBank were found in tissue from symptomatic or asymptomatic pawpaw samples, except for *Phaseolus vulgaris* endornavirus 1 (PvEV1) and *Phaseolus vulgaris* endornavirus 2 (PvEV2), as expected, because the pawpaw leaf tissue was spiked (5%, wt/wt) with leaf tissue of *Phaseolus vulgaris* ‘Black turtle soup’ infected with PvEV1 or PvEV2 prior to total RNA isolation. 

The de novo assembly suggested the presence of TRSV and ToRSV in leaves from pawpaw tree #1 and the sapling and of TRSV in pawpaw tree #2. This was followed up with an analysis of mapped read depth and coverage to the GenBank reference sequences for those viruses (Table 1). For TRSV, the number of mapped reads for RNA1 and RNA2 ranged from 399,818 to 735,306 with a combined coverage ranging from 96% to 97%. Interestingly, the average read depth for RNA2 was consistently around twice that of RNA1 (GenBank accession numbers NC_005096 and NC_005097, respectively) (Table 1). In comparison with TRSV, the number of mapped reads to RNA1 and RNA2 for ToRSV was much smaller (62 to 9253) with a combined coverage ranging from 13% to 31% with the same relative pattern of twice the read depth for RNA2 compared with RNA1 (GenBank accession numbers NC_003839 and NC_003840, respectively) (Table 1). Together, HTS information revealed the occurrence of TRSV and/or ToRSV in symptomatic but not in asymptomatic pawpaw trees and saplings. 

Contigs with lower nucleotide and amino acid similarity to plant viruses for which sequence information is available in GenBank were also identified in the four pawpaw samples. These sequences were triaged for their potential as novel viral agents infecting pawpaw. The most common sequence category consisted of contigs across all four samples that were annotated with similar sequences of viruses in the family *Caulimoviridae*. Since these were present in the asymptomatic pawpaw sample, they were discounted from further consideration. In the category of virus sequences unique to symptomatic pawpaw trees were three contigs in symptomatic pawpaw tree #1 with similar sequences to viruses in the family *Rhabdoviridae*. Rhabdoviruses are known to infect a diverse set of plant species [7]. The three contigs covered all the typical open reading frames for rhabdoviruses. No evidence of this sequence was detected in the other symptomatic pawpaw sample. Therefore, the validation and investigation of a potential novel rhabdovirus sequence was left for future study.

### 2.3. Validation of the Pawpaw Virome and Detection of TRSV and ToRSV

To validate HTS results, leaf samples from the two symptomatic trees #1 and #2 and the sapling selected for HTS, as well as 10 additional trees from the experimental pawpaw orchard were tested for TRSV and ToRSV by RT-PCR using specific primers. In addition, leaf, root, and fruit samples collected from two symptomatic trees in a commercial pawpaw orchard in Westminster, Maryland in June 2022 were similarly tested. Leaf samples from the two asymptomatic trees used in HTS were used as negative controls in RT-PCR. For TRSV, a primer pair designed in the RNA-dependent RNA polymerase (RdRP) coding region was selected for this study (Table 2). For ToRSV, primer pairs designed in the RdRP and coat protein (CP) coding regions, as well as the conserved 3′ untranslated regions (3′-UTRs) of the two genomic RNAs were used in diagnostic RT-PCR (Table 2).

As anticipated, RT-PCR results revealed co-infection by TRSV and ToRSV and single infection by TRSV in the original leaf samples from trees #1 and #2 used for HTS in 2021, respectively. The same results were obtained with new leaf samples collected from these two trees in 2022 (Table 3).

Furthermore, dual infections of TRSV and ToRSV and single infection of TRSV were found in additional leaf samples from 10 trees and saplings collected in the experimental orchard in May 2022. Most leaf samples from trees (75%, 9 of 12) and saplings (77%, 10 of 13) were infected with TRSV (Table 3). ToRSV was only present in dual infection with TRSV in leaf samples of three trees and saplings (Table 3). These results were consistent with a predominance of TRSV and a sporadic presence of ToRSV in the experimental orchard. Interestingly, TRSV was detected in two asymptomatic trees and three asymptomatic saplings, suggesting a latent infection in some trees and saplings or a recovery from disease symptoms (Table 3). Furthermore, TRSV was detected in flowers (83%, 10 of 12) from TRSV-infected trees. Co-infection by TRSV and ToRSV was also detected in flowers of a few trees (17%, 2 of 12) (Table 3). The expected 181 bp amplicon of the internal control *nad5* was obtained in all pawpaw samples in RT-PCR. As anticipated, the two nepoviruses were not detected by RT-PCR in the original asymptomatic samples used for HTS.

Virus diagnosis results based on RT-PCR were confirmed by DAS-ELISA using specific antibodies with a single discrepancy between the two assays: TRSV was found in leaf samples of sapling R2T4 by RT-PCR but not by DAS-ELISA (Table 3). Finally, TRSV was detected by RT-PCR and DAS-ELISA in leaves (100%, 2 of 2), roots (100%, 3 of 3) and fruitlets (100%, 5 of 5) from two symptomatic trees in a commercial pawpaw orchard in Westminster, Maryland (Table 3). Together, the diagnostic work confirmed the presence of TRSV and/or ToRSV in an experimental pawpaw orchard in New York, validating the HTS data, and revealed the occurrence of TRSV in a commercial pawpaw orchard in Maryland.

### 2.4. Association between Disease Symptom Type and Virus Presence

Information on the type of symptoms exhibited by pawpaw trees in the experimental orchard in New York and RT-PCR and DAS-ELISA outputs in a subset of symptomatic trees in this orchard was confronted to determine possible correlations between symptom types and presence of TRSV and/or ToRSV. Results were consistent with no association of specific disease symptoms with one of the two nepoviruses or both viruses, indicating that symptoms of single infections by TRSV or dual infections by TRSV and ToRSV were undistinguishable.

### 2.5. Characterization of TRSV and ToRSV Isolates from Pawpaw

DNA fragments obtained from leaves (trees and saplings) and flowers by RT-PCR for a partial fragment of the RNA1-encoded RdRP coding region of TRSV (320 bp), RNA1-encoded RdRP coding region of ToRSV (580 bp), RNA2-encoded CP coding region of ToRSV (668 bp), and for the 3′-UTRs (480 bp) of ToRSV were characterized by Sanger sequencing to assess the genetic relatedness among pawpaw isolates and their relationship with TRSV and ToRSV isolates from other crops for which sequence information is available in GenBank. The partial TRSV and ToRSV sequences from pawpaw isolates that were determined in this study by RT-PCR and Sanger sequencing were deposited in GenBank as accession numbers OP806051-OP806076. 

Sequence data analyses revealed high nucleotide sequence identities (94.9–100%) in the RdRP coding region among TRSV isolates from six symptomatic and two asymptomatic pawpaw trees and saplings, and between pawpaw isolates and isolates from other crops (93.6–100%) (Table 4). High nucleotide sequence identities (96.9–99.3%) were also obtained in the RdRP coding region among ToRSV isolates from three pawpaw isolates, and between pawpaw isolates and isolates from other crops (81.6–98.9%) (Table 4). In the partial ToRSV CP fragment, the nucleotide sequence identity was high among isolates from pawpaw (91.8–100%), and between pawpaw isolates and isolate DSMZ PV-0381 from grapevine (89.6–96%) but less between pawpaw isolates and isolates from other crops (71.3–83.9%) (Table 4). In the partial ToRSV RNA1 3′-UTR, the nucleotide sequence identity was high among ToRSV isolates from pawpaw (98–100%), and between pawpaw isolates and isolates DSMZ PV-0381 from grapevine and Rasp1-2014 from raspberry (97.8–99.3%) but less between pawpaw isolates and those from other crops (79.2–87.8%) (Table 4). Similarly, in the partial ToRSV RNA2 3′-UTR, the nucleotide sequence identity between pawpaw isolates and isolates DSMZ PV-0381 and Rasp1-2014 was high (87–99.3%) but less between pawpaw isolates and isolates from other crops (79.4–88%) (Table 4). 

## 3. Discussion

We identified and characterized TRSV and ToRSV by HTS in pawpaw trees exhibiting stunting and/or foliar patchy discolorations, chlorosis, mottling, vein clearing, and distortions in an experimental orchard at Cornell University. The presence of TRSV and ToRSV was validated in the trees from which nucleic acids were isolated for HTS and in additional trees from the same experimental orchard by RT-PCR and DAS-ELISA. Although we did not attempt to demonstrate causality, it is reasonable to presume that TRSV in single infection or TRSV and ToRSV in dual infections are responsible for the symptoms observed in the experimental pawpaw orchard at Cornell University, given the high association between the occurrence of one or both viruses and disease symptoms in the pawpaw trees tested, as well as the history of the pawpaw trees. Additionally, TRSV was found by RT-PCR and DAS-ELISA in symptomatic pawpaw trees in a commercial orchard in Maryland.

TRSV and ToRSV are both transmitted non-persistently by ectoparasitic dagger nematode vectors of the *Xiphinema americanum* group [12]. The almost even distribution of symptomatic trees throughout the experimental orchard at Cornell University, except at the edge at the north and south sides although some of these trees were infected with TRSV, suggested that most of the trees were likely infected with TRSV when the orchard was established. If *X*. *americanum* nematodes would be involved in the spatiotemporal distribution of this virus in the experimental orchard, patchy aggregations of symptomatic trees would be expected. This was not the case because the distribution of symptomatic trees was non-clustered. In addition, the fact that some of the asymptomatic trees were infected with TRSV suggested that the planting material rather than dagger nematode vector-mediated virus transmissions likely explains the widespread distribution of TRSV in the experimental orchard. Nonetheless, we cannot rule out the presence of *X*. *americanum* in the experimental orchard and their involvement in short distance spread of TRSV or even ToRSV. Soil samples would need to be collected at various sites and tested for *X*. *americanum* to ascertain the occurrence of this nematode vector in the experimental pawpaw orchard.

Validating efforts of HTS results revealed TRSV in every leaf sample tested from symptomatic and asymptomatic trees in the experimental pawpaw orchard at Cornell University (Table 3). This finding supports the idea that trees were likely infected with TRSV when the orchard was established. It is possible that the TRSV infection was latent initially, and disease symptoms became progressively apparent after the orchard was established. Viruses switching from latency to a disease symptom-causing state are occasionally observed in infected perennial crops due to various factors including environmental stimuli, developmental growth stage, and mixed virus infections [13]. Additionally, it can be speculated that, given TRSV is pollen and seed transmitted in several crops such as soybean and *Pelargonium* hybrids among others [14,15,16,17,18], the pawpaw seedlings used as rootstock for producing the experimental trees were initially infected. The fact that TRSV was identified in different grafted pawpaw cultivars in distant orchards in New York and Maryland adds credence to the rootstock seedling origin of the virus. Similarly, the detection of TRSV in flowers collected in May 2022 from infected trees in the experimental orchard in New York, and in fruitlets sampled in the commercial orchard in Maryland in June 2022, adds plausibility to the virus transmissibility via pollen and/or seed in pawpaw; however, these hypotheses need to be experimentally tested for validation.

The detection of ToRSV was sporadic in the experimental orchard at Cornell University, suggesting its possible presence in some but not all the propagation material used to produce the pawpaw trees. A more plausible explanation for the presence of ToRSV in the experimental orchard site is that this virus occurred in alternative plant hosts, likely in weeds, prior to the establishment of the pawpaw trees. Then, *X*. *americanum*-mediated ToRSV transmission may have occurred from infected weeds to some pawpaw trees and saplings. This hypothesis is further supported by the fact that ToRSV was not identified in symptomatic leaf samples from a pawpaw orchard in Maryland where the virus may not be present. Interestingly, dual infections by TRSV and ToRSV were detected in leaf samples of tree #1 and its sapling, and in flowers of tree #2 (Table 3). This result may suggest that ToRSV infection in flowers of tree #2 may have resulted from pollen-mediated inoculation.

Sequence analyses following HTS and Sanger sequencing of RT-PCR amplicons revealed a close genetic relatedness of TRSV and ToRSV isolates from pawpaw based on a high nucleotide sequence identity in a partial RdRP fragment (94.9–100% and 96.9–99.3%, respectively). A similar high nucleotide sequence identity was found between pawpaw isolates and isolates from other crops (96.3–100% for TRSV and 81.6–98.9% for ToRSV). For the partial CP fragment and 3′-UTR of ToRSV, the nucleotide sequence identity was high among isolates from pawpaw (91.8–100% and 98–100%, respectively) but lower between pawpaw isolates and isolates from other crops (71.3–96% and 79.2–99.3%). Nonetheless, the percent nucleotide sequence identities determined for the ToRSV isolates from pawpaw in the partial RdRP, CP, and 3′-UTR fragments fall within the ranges previously reported (78–100%) [10,19]. 

Pathogens of pawpaw are few because they are based on limited accounts in the literature. Declining pawpaw trees were reported in the late 1990s from Oregon. A disease origin of the decline was suspected but no pathogen, particularly no fungi or bacteria, was consistently isolated from declining trees [20]. Curiously, viruses were not investigated in this work. Our study is the first to ever report the occurrence of a pathogen in symptomatic pawpaw trees with the identification of ToRSV and TRSV. The fact that these two nepoviruses were found in symptomatic trees and some of their saplings was consistent with the tissue connectivity of the tree and its rhizome, explaining the dual infection in the two types of tissues through systemic infection. Similarly, the detection of TRSV in root and fruit tissue of some trees in the pawpaw orchard in Maryland confirms a systemic infection. The recovery from symptoms in pawpaw tree is also consistent with the occurrence of TRSV and ToRSV, two nepoviruses for which initial infection causes severe systemic symptoms and infected plants recover later, as documented on experimental herbaceous plant hosts [21,22,23]. 

The pawpaw is a delicious and nutritious native American fruit. A recent increased interest in pawpaw amongst groups of Indigenous Peoples, gardeners, and nontraditional fruit enthusiasts has driven up the demand for pawpaw fruits and trees [4,5]. The propagation of clonal pawpaw rootstocks has been unsuccessful; therefore, nurseries currently graft cultivars onto rootstock derived from locally available seeds of diverse genetic origin. Alternatively, nurseries sell dormant or sprouted seeds [24,25]. Pawpaw orcharding is advertised as a business opportunity for small growers, particularly as food processors develop recipes to turn mature pawpaw fruit into value-added products [26,27]. Beyond North America, pawpaw plantings are documented in Romania, Italy, Ukraine, Slovakia, Austria, Georgia, Russia, Japan, and Korea [28,29]. Some of these plantings are derived from sprouted seeds, whereas others are planted with grafted cuttings [30]. 

There is no cure for TRSV and ToRSV once trees are infected in the orchard. Therefore, based on the expansion of pawpaw orchards in the United States and worldwide, a careful selection of clean pawpaw seeds and propagation material is critical for producing clean grafted cultivars and preventing the introduction of viruses, such as TRSV and ToRSV, in newly established orchards. Such simple measures are salient for pawpaw because this deciduous fruit tree is considered vulnerable or endangered in several regions of the United States, and there is a strong desire to save this indigenous fruit tree species by Indigenous Peoples in New York.

## 4. Materials and Methods

### 4.1. Experimental Pawpaw Orchard

An experimental pawpaw orchard was planted in 1999 on a research farm at Cornell University in Lansing, New York, USA. The orchard consisted of 28 pawpaw selections, including 10 commercial cultivars (Middletown, Mitchell, NC-1, Overleese, PA-Golden #1, Sunflower, Taylor, Taytwo, Wells, and Wilson), and 18 advanced open-pollinated seedling lines. Each selection was planted as eight replicates in four rows of 56 trees each. The between- and within-row spacing was 5.2 m and 1.8 m, respectively. The orchard is located on an 8% southwestern slope on gravelly silt loam soil with a pH ranging from 5.6 to 6.2. No pesticide or herbicide was ever applied. Beneath the pawpaw trees, numerous pawpaw saplings arose from either rhizomes or seeds that sprout from unharvested fruit dropping from the tree branches. No attempt was made in this study to distinguish saplings originating from rhizomes or seeds.

### 4.2. Visual Assessment of Virus-like Symptoms in the Experimental Pawpaw Orchard

Individual trees and surrounding saplings were monitored for virus-like symptoms in the experimental pawpaw orchard in May 2022 to assess the incidence and distribution of symptomatic trees. Symptoms of tree stunting, foliar discolorations, chlorosis, mottling, vein clearing, and distortions were recorded.

### 4.3. Pawpaw Plant Material

Six to eight pawpaw leaves (10–20 cm in length) from the mid-point of branches were collected in October 2021 from symptomatic and asymptomatic pawpaw trees, as well as from symptomatic pawpaw saplings growing underneath the trees in the experimental orchard managed by Cornell University. Leaf samples were similarly collected from two asymptomatic pawpaw trees located on the main campus of Cornell University in Ithaca, New York, which is approximately 12 km away from the experimental orchard. These samples were selected as negative controls in HTS work to avoid confounding effects of possible virus infections in asymptomatic trees from the experimental orchard. Additional leaf samples were collected from the same pawpaw trees, and eight additional symptomatic and two asymptomatic trees in the experimental orchard in May 2022 for validation work. Finally, flowers were collected from those 12 selected trees in the experimental pawpaw orchard for virus testing.

Roots, flowers, small fruits, and symptomatic leaves were also collected from two grafted ‘Shenandoah’ trees in a commercial pawpaw orchard in Westminster, Maryland in June 2022 for virus testing. 

### 4.4. Total Nucleic Acid Isolation from Pawpaw Leaves and HTS

Total nucleic acid extracts were prepared from symptomatic and asymptomatic pawpaw leaf tissue, as previously described [31]. Briefly, approximately 0.2 g of pawpaw leaf tissue was spiked with leaf tissue of black turtle soup dry bean plants infected by PvEV1 and PvEV2 (5%, wt:wt), and homogenized using a Homex grinder (Bioreba, Reinach, Switzerland). Total nucleic acid extracts were prepared using a MagMAX-96 viral RNA isolation kit (Thermo Fischer Scientific, Waltham, MA, USA), as per the manufacturer’s protocol. Aliquots of total nucleic acid samples were subjected to ribosomal RNA depletion and a complementary DNA (cDNA) library construction was built using a TruSeq Stranded Total RNA with Ribo-Zero Plant kit (Illumina, San Diego, CA, USA). Libraries were run on an Illumina NextSeq 500 instrument using a 75-bp single-end read platform.

### 4.5. HTS Data Analysis

Illumina sequence reads were adapter trimmed and, subsequently, de novo assembled into contigs of at least 200 bp in length using the SPAdes (v. 3.14) [32]. Contigs were then annotated to generate viral sequence candidates. The initial list of contigs was generated using the BLASTx and BLASTN programs (v. 2.4.0) [33] to compare contig sequences against the National Center for Biotechnology Information GenBank Database (https://www.ncbi.nlm.nih.gov/genbank/, accessed on 16 December 2021). All contigs matching plant viral genomes with a combined E-value equal to or less than 10^−4^ were considered candidates of interest. For virus positives, reads counts, average depth, and bases covered were computed for all RNAs by mapping reads to the GenBank reference sequences using Bowtie2 [34] and SAMtools [35].

### 4.6. Validation of HTS Results by RT-PCR and Sanger Sequencing

HTS results were validated by one-step reverse transcription (RT)-polymerase chain reaction (PCR) using the OneStep *Ahead* RT-PCR kit (Qiagen, Germantown, MD, USA) and specific TRSV and ToRSV primers (Table 2). A primer pair (5′-GATGCTTCTTGGGGCTTCTTGTT-3′ and 5′-CTCCAGTCACCAACATTGGCATAA-3′) specific to the 181 bp fragment of the nicotinamide adenine dinucleotide coenzyme dehydrogenase subunit 5 (*nad5*) gene was used as an internal control [36]. Total RNA was extracted from pawpaw leaf or flower tissue (100 mg) using the MagMAX-96 viral RNA isolation kit (Thermo Fischer Scientific, Waltham, MA, USA) after processing with a TissueLyserII (Qiagen, Germantown, MD, USA). Thermocycling conditions were 1 cycle of 50 °C for 10 min and 95 °C for 5 min; 40 cycles of 95 °C for 10 s, 55 °C for 20 s, and 72 °C for 20 s; and 1 cycle of 72 °C for 2 min. The annealing temperature for the second step of the 40 cycles varied with the primer pair (Table 2). The amplified products were resolved by electrophoresis on 2% agarose gels in Tris-acetate-EDTA buffer and visualized under UV light after straining with GelRed^®^ (Biotium, Fremont, CA, USA). For some pawpaw samples, RT-PCR amplicons were treated with ExoSAP-IT (Thermo Fischer Scientific, Waltham, MA, USA) and sequenced in both directions for verification of DNA integrity by Sanger sequencing at the Cornell Biotechnology Resource Center. 

The nucleotide sequences of TRSV and ToRSV obtained in this study were trimmed and assembled using the RNA1 sequence of TRSV isolate SK (GenBank accession number KJ556849), and RNA1 (GenBank accession number KM083894) and RNA2 (GenBank accession number KM083895) sequences of ToRSV isolate Rasp1-2014 as references. Analyses of the TRSV and ToRSV sequences were performed using ClustalW in the MegAlign software of DNASTAR Lasergene, and included several sequences retrieved from GenBank (Table 4).

### 4.7. Validation of HTS Results by DAS-ELISA

In addition to RT-PCR, HTS results were also validated by DAS-ELISA with specific antibodies (Bioreba, Reinach, Switzerland). A portion of 4–6 stacked pawpaw leaves was torn, ground in phosphate buffer saline supplemented with 10 mM sodium sulfite, 1% polyvinylpyrrolidone 40, 1% Tween 20 and powdered egg albumin (2 g/L) at a 1:10 ratio (*w*:*v*) using a semi-automated ball-bearing HOMEX tissue homogenizer (Bioreba, Reinach, Switzerland), and tested for TRSV and ToRSV according to the manufacturer’s instructions. Substrate hydrolysis was recorded at 405 nm with an absorbance BioTek ELx808TM microplate reader (BioTek, Winooski, VT, USA). Samples were considered positive if their mean optical density (OD_405nm_) readings were at least twice those of healthy controls.

## 5. Conclusions

TRSV and ToRSV were identified by HTS in an experimental pawpaw orchard at Cornell University in New York. HTS findings were validated by RT-PCR using specific primers in combination with Sanger sequencing, and by DAS-ELISA with specific antibodies. Single infections by TRSV were predominant in the pawpaw orchard while co-infections by TRSV and ToRSV were sporadic. No single infection by ToRSV was detected. The prevalence of TRSV suggested an introduction of this virus in the experimental orchard via the propagation material, likely through infected rootstock seedlings. This hypothesis is validated by the identification of TRSV in symptomatic trees in a commercial pawpaw orchard in Maryland. In contrast, the sporadic presence of ToRSV in the experimental pawpaw orchard may have resulted from transmission by *Xiphinema americanum* dagger nematodes from infected weeds. To our knowledge, this study is the first to determine the virome of pawpaw and identify two soil-borne viruses in pawpaw trees.

## Figures and Tables

**Table 1:** Summary of sequencing and mapping statistics from HTS data.

Sample	Total Reads ^a^	Virus Identified ^b^	No. Viral Reads ^c^	RPKM ^d^	% Viral Reads ^e^	% Viral Gen. Cov. ^f^	Virus Contigs ^g^	% Identity ^h^
R1T13 tree	23,669,589	TRSV + ToRSV	402,568 + 9253	1486 + 25	1.7	97.8 + 30.9	45 + 5	99 + 95
R1T13 sapling	16,215,059	TRSV + ToRSV	735,306 + 62	3963 + 0.2	4.5	96.0 + 13.3	34 + 4	99 + 96
R2T5	24,315,199	TRSV	398,818	2154	2.5	97.4 + 0	62 + 0	99
Control	20,731,853	None	None	0	0	0 + 0	0 + 0	0

^a^ Total number of reads per pawpaw sample. ^b^ Tobacco ringspot virus (TRSV) and tomato ringspot virus (ToRSV). ^c^ Total number of unique reads mapped to viral reference sequences for TRSV and ToRSV, respectively. ^d^ RPKM: Reads per kilobase of viral transcript per million mapped reads for TRSV and ToRSV, respectively. ^e^ Percentage of viral reads from total number of reads obtained per sample. ^f^ Viral genome coverage values are indicated for TRSV and ToRSV, respectively. ^g^ Numbers of de novo assembled contigs for TRSV and ToRSV, respectively. ^h^ BLASTN percentage identity between de novo assembled viral genome and the best-hit viral genome for TRSV and ToRSV, respectively.

**Table 2:** List of diagnostic primers used to detect tobacco ringspot virus (TRSV) and tomato ringspot virus (ToRSV) by RT-PCR in pawpaw samples.

Virus	Coding Region ^a^	Primer Sequence	Amplicon (bp) ^b^	T (°C) ^c^	Reference
TRSV	RdRP	5′-CAATACGGTAAGTGCACACCCCG-3′	320	59	[8]
		5′-CAGGGGCGTGAGTGGGGGCTC-3′			
ToRSV	RdRP	5′-CCACCACACTCCACCTACC-3′	580	58	[8]
		5′-ACTTCTGAAGGCTACCCGTT-3′			
	CP	5′-GTTCCTGCGGAAGCTGATTG-3′	668	55	[9]
		5′-GGCCACTCATACCTCCAGTC-3′			
	3′-UTR	5′-AGGTAGGACGCYATTGTTCCAGG-3′	480	51	[10]
		5′-AGTCTCAACTTAACATACCACTAC-3′			

^a^ RdRP: RNA-dependent RNA polymerase; CP: coat protein, 3′-UTR: 3′ untranslated region. ^b^ Expected size of the RT-PCR amplicons. ^c^ Annealing temperature.

**Table 3:** Detection of tobacco ringspot virus (TRSV) and tomato ringspot virus (ToRSV) by RT-PCR and DAS-ELISA in leaves or flowers of pawpaw trees and saplings collected from an experimental orchard and a commercial orchard.

			Positive for					
			TRSV + ToRSV ^f^			TRSV		
Orchard ^a^	Symptoms ^e^	N	Tree	Sapling	Flower	Tree	Sapling	Flower
Experimental								
R1T2	yes	3	0	0	0	1	1	1
R1T13 ^b^	yes	3	1	1	0	0	0	1
R1T15	yes	3	0	0	0	1	1	1
R2T4 ^c^	yes	3	0	1	0	1	0	1
R2T5 ^b^	yes	3	0	0	1	1	1	0
R2T11	yes	3	1	0	0	0	1	1
R3T1	no	4	0	0	0	1	2	1
R3T3	yes	3	0	0	0	1	1	1
R4T8	yes	3	0	0	0	1	1	1
R4T16	yes	3	1	1	1	0	0	0
R4T26	yes	3	0	0	0	1	1	1
R4T33	no	3	0	0	0	1	1	1
Commercial ^d^								
T1	yes	1	0	nt	nt	1	nt	nt
T2	yes	1	0	nt	nt	1	nt	nt
Total		39	3	3	2	11	10	10

^a^ Leaf samples collected from pawpaw trees in an experimental orchard at Cornell University. The location of the trees is indicated by a row number (R) and a within-row position (T). ^b^ Leaves from this tree (R1T13, tree #1) and sapling (R2T5, tree #2) were used for HTS. ^c^ Sample for which a discrepancy between RT-PCR (positive) and DAS-ELISA (negative) was obtained for TRSV. ^d^ Leaves collected from two symptomatic pawpaw trees in a commercial orchard in Maryland. ^e^ Virus-like symptoms of tree stunting, and foliar mosaic, vein clearing, chlorosis, and deformations were recorded in May 2022. ^f^ Dual infection by TRSV and ToRSV is represented as “TRSV + ToRSV”, and single infection by TRSV is represented as “TRSV”. nt: not tested.

**Table 4:** Nucleotide sequence identities of tobacco ringspot virus (TRSV) and tomato ringspot virus (ToRSV) isolates from pawpaw trees and other crops in the RNA-dependent RNA polymerase (RdRP), coat protein (CP), and 3′ untranslated (3′-UTR) genomic regions.

Virus	Isolate ^a^	Crop ^b^	Genome	Accession No.^c^	Sequence Identity (%) ^d^		
					RdRP	CP	3′-UTR
TRSV	CmTX-M	Melon	RNA1	MN504771	93.6–97.3	na	nt
	DSMZ PV-0236	Common bean	RNA1	MW057706	95.9–98.6	na	nt
	SK	Soybean	RNA1	KJ556849	96.3–100.0	na	nt
	YW	Japanese water iris	RNA1	MT042825	96.3–98.0	na	nt
	WA-AM1	Blueberry	RNA1	MW495243	95.6–99.3	na	nt
ToRSV	13C280	Prunus *	RNA1	KM083890	86–86.2	na	86.5–87.8
			RNA2	KM083891	na	82.8–83.9	86.8–88.0
	DSMZ PV-0381	Grapevine	RNA1	MW057702	88.4–89.2	na	97.8–99.3
			RNA2	MW057703	na	89.6–96.0	97.8–99.3
	GYV-2014	Grapevine *	RNA1	KM083892	81.6–82.0	na	79.2–80.4
			RNA2	KM083893	na	71.3–71.6	79.4–80.6
	Rasp1-2014	Raspberry *	RNA1	KM083894	96.5–98.9	na	97.8–99.3
			RNA2	KM083895	na	81.7–82.5	87–88.3

^a^ TRSV and ToRSV isolates used in this study for the sequence analyses. ^b^ Crop host of virus isolates. ^c^ GenBank accession number. ^d^ Nucleotide sequence identity of TRSV and ToRSV isolates from pawpaw and isolates for which information was retrieved from the NCBI GenBank database. * ToRSV isolates maintained in *Nicotiana benthamiana* (GYV-2014 and Rasp1-2014) and *Prunus tomentosa* and GF305 peach (13C280) [11]. nt: not tested; na: not applicable.

## Data Availability

The sequences of TRSV and ToRSV isolates from pawpaw that were determined in this study are available in GenBank as accession numbers OP806051-OP806076.
